# Where boundaries become bridges: Mosquito community composition, key vectors, and environmental associations at forest edges in the central Brazilian Amazon

**DOI:** 10.1371/journal.pntd.0011296

**Published:** 2023-04-26

**Authors:** Adam Hendy, Eduardo Hernandez-Acosta, Danielle Valério, Nelson Ferreira Fé, Claudia Reis Mendonça, Edson Rodrigues Costa, Eloane Silva de Andrade, José Tenaçol Andes Júnior, Flamarion Prado Assunção, Vera Margarete Scarpassa, Marcus Vinícius Guimarães de Lacerda, Michaela Buenemann, Nikos Vasilakis, Kathryn A. Hanley

**Affiliations:** 1 Department of Pathology, University of Texas Medical Branch, Galveston, Texas, United States of America; 2 Department of Biology, New Mexico State University, Las Cruces, New Mexico, United States of America; 3 Instituto de Pesquisa Clínica Carlos Borborema (IPCCB), Fundação de Medicina Tropical Doutor Heitor Vieira Dourado (FMT-HVD), Manaus, Amazonas, Brazil; 4 Centro de Entomologia, Fundação de Medicina Tropical Doutor Heitor Vieira Dourado (FMT-HVD), Manaus, Amazonas, Brazil; 5 Laboratório de Biologia da Conservação, Projeto Sauim-de-Coleira, Instituto de Ciências Biológicas, Universidade Federal do Amazonas, Manaus, Amazonas, Brazil; 6 Coordenação de Biodiversidade, Instituto Nacional de Pesquisas da Amazônia, Manaus, Amazonas, Brazil; 7 Instituto Leônidas and Maria Deane, Fiocruz Amazonas, Manaus, Amazonas, Brazil; 8 Department of Geography and Environmental Studies, New Mexico State University, Las Cruces, New Mexico, United States of America; 9 Center for Biodefense and Emerging Infectious Diseases, University of Texas Medical Branch, Galveston, Texas, United States of America; 10 Institute for Human Infection and Immunity, University of Texas Medical Branch, Galveston, Texas, United States of America; 11 Center for Tropical Diseases, University of Texas Medical Branch, Galveston, Texas, United States of America; 12 Center for Vector-Borne and Zoonotic Diseases, University of Texas Medical Branch, Galveston, Texas, United States of America; University of Oxford, UNITED KINGDOM

## Abstract

Risk of spillover and spillback of mosquito-borne viruses in the neotropics, including yellow fever, dengue, Zika (*Flaviviridae*: *Flavivirus*), chikungunya, and Mayaro (*Togaviridae*: *Alphavirus*) viruses, is highest at ecotones where humans, monkeys, and mosquitoes coexist. With a view to identifying potential bridge vectors, we investigated changes in mosquito community composition and environmental variables at ground level at distances of 0, 500, 1000, and 2000 m from the edge of a rainforest reserve bordering the city of Manaus in the central Brazilian Amazon. During two rainy seasons in 2019 and 2020, we sampled 9,467 mosquitoes at 244 unique sites using BG-Sentinel traps, hand-nets, and Prokopack aspirators. Species richness and diversity were generally higher at 0 m and 500 m than at 1000 m and 2000 m, while mosquito community composition changed considerably between the forest edge and 500 m before stabilizing by 1000 m. Shifts in environmental variables mainly occurred between the edge and 500 m, and the occurrence of key taxa (*Aedes albopictus*, *Ae*. *scapularis*, *Limatus durhamii*, *Psorophora amazonica*, *Haemagogus*, and *Sabethes*) was associated with one or more of these variables. Sites where *Ae*. *aegypti* and *Ae*. *albopictus* were detected had significantly higher surrounding mean NDBI (Normalized Difference Built-up Index) values than sites where they were not detected, while the opposite was true for *Sabethes* mosquitoes. Our findings suggest that major changes in mosquito communities and environmental variables occur within 500 m of the forest edge, where there is high risk for contact with both urban and sylvatic vectors. By 1000 m, conditions stabilize, species diversity decreases, and forest mosquitoes predominate. Environmental variables associated with the occurrence of key taxa may be leveraged to characterize suitable habitat and refine risk models for pathogen spillover and spillback.

## Author summary

Mosquito-borne viruses, including yellow fever, dengue, Zika, chikungunya, and Mayaro viruses, derive from sylvatic transmission cycles involving forest mosquitoes and wild animals. Occasionally, these sylvatic viruses spill over into humans, which can lead to limited disease outbreaks or sustained human transmission by urban mosquitoes. When translocated by humans to new geographic regions, these viruses have potential to spill back into novel sylvatic cycles, creating a lasting threat to human health. Ecotones, such as forest edges, where humans, mosquitoes, and monkeys coexist, are at high risk of spillover and spillback. We investigated changes in mosquito community composition at incremental distances up to 2000 m into rainforest bordering the Amazonian city of Manaus, where the above viruses circulate in urban and sylvatic cycles. We also characterized mosquito habitat and analyzed associations between key vector species and environmental variables like temperature and vegetation. Our results show that mosquito communities and environmental variables change abruptly within 500 m of the forest edge, where there is high risk for contact of both monkeys and humans with both urban and forest mosquitoes. By 1000 m, conditions stabilize, and forest mosquitoes predominate. Environmental variables associated with the presence of particular mosquito taxa will help characterize suitable habitat for key vector species and refine spillover and spillback risk models for these viruses.

## Background

The potential for pathogen exchange between native and novel hosts is thought to be highest at ecosystem boundaries [1,2]. The risk of such exchange is particularly acute for arthropod-borne viruses (arboviruses), as all arboviruses affecting humans derive from wildlife transmission cycles [3]. Arboviruses transmitted by mosquitoes have the greatest public health impact, and the most prominent of these are transmitted in sylvatic cycles between non-human primates (monkeys) and mainly canopy-dwelling mosquitoes [4], including yellow fever (YFV), dengue (DENV), Zika (ZIKV) (all *Flaviviridae*: *Flavivirus*), and chikungunya (CHIKV) (*Togaviridae*: *Alphavirus*) [5]. Spillover of these viruses from their sylvatic cycles, mediated by mosquito bridge vectors, can lead to isolated human infections, localized epidemics, or establishment of human endemic cycles with global reach [3].

Of all the mosquito-borne viruses, the role of ecotones in spillover has been most firmly established for YFV [1]. This virus emerged from its ancestral African sylvatic cycle and was introduced to the Americas approximately 400 years ago [6], where it soon spilled back into a novel sylvatic cycle involving neotropical monkeys and *Haemagogus* and *Sabethes* species mosquitoes. In recent decades, DENV, ZIKV, and CHIKV have also emerged or resurged in the Americas where, up to now, they have been transmitted exclusively in urban cycles by domestic *Aedes aegypti* and peridomestic *Ae*. *albopictus* mosquitoes [3,4]. In areas where humans, monkeys, and mosquitoes coexist, these viruses could potentially spill back into novel sylvatic cycles which may be impossible to eradicate, thereby creating a lasting threat to human health [7–9].

While it is widely accepted that risk of arbovirus spillover and spillback is elevated at forest edges, the mechanisms for such effects are incompletely understood. Borremans et al. [2] posit that the “permeability” of edges for mosquito-borne arbovirus exchange will depend upon the abundance, biting rates, and competence of mosquito vectors, as well as host abundance, near edges. Moreover, edge effects on microclimate are likely to alter the ability of a given mosquito to transmit an arbovirus [10], with warmer forest edges likely enhancing vector competence. Studies investigating shifts in mosquito communities at forest edges often utilize mechanized sampling methods [11–13], delivering a biased view of mosquito diversity and abundance and no insight into biting rates. For example, we previously used BG-Sentinel traps and Prokopack aspirators to sample mosquitoes at ground level in urban forest fragments in Manaus, Brazil [13]. These aspirators are designed to sample resting, potentially blood fed mosquitoes [14], which are not necessarily anthropophilic. In contrast, BG-Sentinel traps are designed to sample host-seeking *Aedes* and *Culex* species mosquitoes [15] but are less specific than humans in attracting anthropophilic mosquitoes [16]. Consequently, the choice of sampling method skews collections towards mosquitoes attracted to associated stimuli. For instance, anthropophilic mosquitoes may be attracted to CO_2_ and other attractants associated with BG-Sentinel traps, but so might ornithophilic species, whereas ornithophilic species would likely not be attracted to a human wielding a hand-net.

In our earlier study of urban forest fragments [13], *Ae*. *albopictus* was the most abundant urban vector species sampled. It occurred most frequently at forest edges but penetrated at least 100 m into the forest. We have since used BG-Sentinel traps and hand-nets [17,18] to investigate vertical changes in mosquito communities at the Adolpho Ducke forest reserve bordering Manaus, showing that *Haemagogus* and *Sabethes* mosquitoes, along with *Psorophora amazonica*, could potentially provide routes for pathogen exchange between the forest canopy and forest floor. Our studies have shown BG-Sentinel traps to be effective for the collection of urban *Aedes* vectors and *Psorophora* species among others, while hand-net collections have yielded greater numbers of *Haemagogus* mosquitoes including *Hg*. *janthinomys* [13,17]. Both methods have proven reasonably effective for the collection of *Sabethes* species mosquitoes [17].

In this study, we investigated ground-level changes in mosquito community composition at 0, 500, 1000, and 2000 m into the forest at the same reserve, using both mechanized and human-operated sampling methods, with a view to identifying potential bridge vectors of mosquito-borne viruses. We predicted that urban species, including known *Aedes* vectors, would predominate at edge sites, and that considerable changes in species composition would occur between 0 m and 500 m, consistent with our previous study of interior forest parks in Manaus [13]. Beyond 500 m, we predicted that collections would be dominated by sylvatic species. Furthermore, we anticipated that the occurrence of *Haemagogus* and *Sabethes* mosquitoes would increase with increasing temperature and decreasing relative humidity, but *Psorophora* mosquitoes would show no such associations, in broad agreement with our previous findings [17,18].

## Methods

### Ethics statement

Mosquito collections were approved by local environmental authorities (SISBIO license 57003–6) and the study did not involve endangered or protected species. When sampling with hand-nets, skin was not deliberately exposed to attract mosquitoes and mosquito landing was not permitted. All collectors are listed among the co-authors or acknowledgements and were fully aware of the nature of the research. They wore trousers, a long-sleeved shirt and/or repellent to minimize the risk of being bitten and had been vaccinated against yellow fever.

### Study area

**Fig 1:**
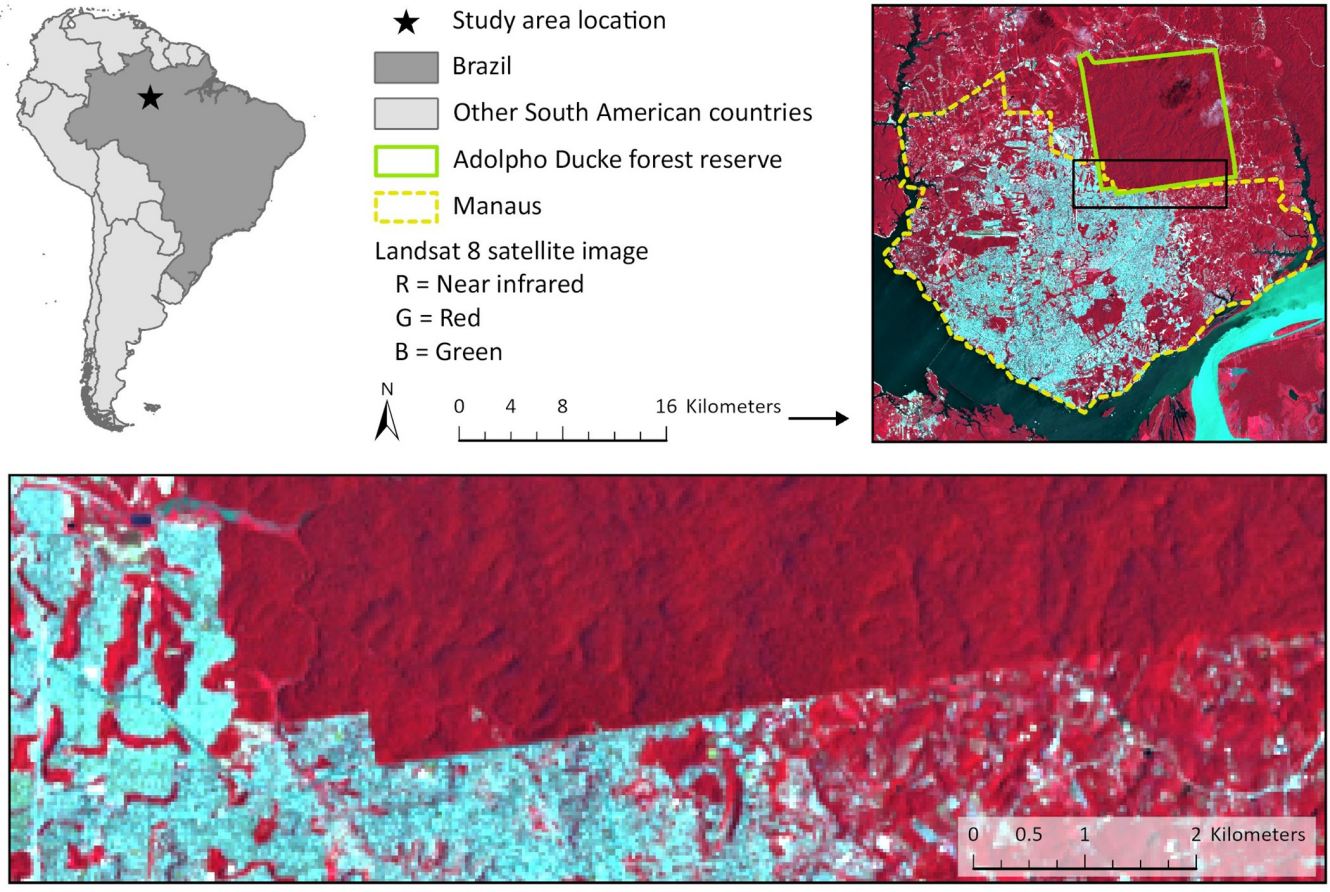
Reference map of the study area. The upper left panel shows the study location in Manaus, Brazil, South America. The other panels show Manaus, the Adolpho Ducke forest reserve, and the edge between city and forest superimposed on a color infrared composite based on Landsat 8 Operational Land Imager surface reflectance imagery obtained from the USGS Earth Explorer data portal [24]. The composite was generated by placing the near infrared, red, and green spectral bands of the satellite image in the red, green, and blue color channels of the computer system. Red areas represent healthy green vegetation; cyan areas represent built-up surfaces and the sediment-laden Amazon River (Rio Solimões) which confluences with the nearly sediment-free Rio Negro at the Meeting of Waters (Encontro das Águas) south of Manaus. Country boundaries obtained from: t als://www.naturalearthdata.com/downloads/10m-cultural-vectors/10m-admin-0-countries/.

The Adolpho Ducke forest reserve (Ducke) encompasses 100 km^2^ of primary rainforest which borders urban Manaus along the northeastern edge of the city [17,18] (Fig 1). Ducke is mainly accessed by researchers and students and is home to six monkey species: the golden-faced saki (*Pithecia chrysocephala*), pied tamarin (*Saguinus bicolor*), tufted capuchin (*Sapajus apella*), Guianan red howler monkey (*Alouatta macconnelli*), Guiana spider monkey (*Ateles paniscus*), and bearded saki (*Chiropotes chiropotes*) [19]. A grid-like network of trails arranged at 1 km intervals and covering 64 km^2^ allows access to much of the reserve. Manaus has undergone rapid urban expansion since the 1980s due to government incentives to develop the region, which has led to deforestation and brought people into close contact with forest edges [20]. This is especially pertinent along the southern and western edges of the reserve where dense urban areas touch the forest [19]. DENV, ZIKV, and CHIKV circulate in Manaus during the rainy season [21], which usually lasts from November to May [19]. Both YFV [22] and Mayaro virus (MAYV) (*Togaviridae*: *Alphavirus*) [23] are known to occur in rainforest near the city.

### Remote sensing environmental variables

Three variables were derived from remote sensing data: land cover, Normalized Difference Vegetation Index (NDVI), and Normalized Difference Built-up Index (NDBI). NDVI and NDBI have theoretical values ranging between -1 and +1 with those > 0 representing vegetated and built-up areas, respectively [25,26]. Both indices are potentially useful for predicting mosquito vector occurrence [26,27]. The remotely sensed data were obtained from the USGS Earth Explorer data portal [24] and included Landsat 8 Operational Land Imager surface reflectance imagery acquired over the study area on 30 July 2017, the most cloud free image acquisition date close to the time of field data collection. Five major land cover types (urban/built-up land, forest land, grassland/agricultural land, barren land, water) were mapped using the random forest classifier at a spatial resolution of 30 m. To define sampling strata, NDVI values were derived for forest land pixels and classified into low (0.472 to 0.859), medium (0.860 to 0.869), and high (0.870 to 1.000) NDVI value categories as described previously [13]. NDBI values were derived for urban/built-up land pixels and classified using the Jenks natural breaks method into low (-1 to -0.467), medium (-0.468 to -0.098), and high (-0.099 to 1.000) NDBI value categories. Several additional NDBI-related variables were calculated to investigate associations between the occurrence of mosquito species and built-up areas. These were the distance from each sampling site to the nearest low, medium, and high NDBI pixel; the maximum NDBI value in the 3 x 3-pixel neighborhood around each site, and the mean NDBI value within 50, 100, 500, 1000, and 2000 m radius around each site. All geospatial analyses were conducted in ArcGIS Pro (ESRI, Redlands, California), ENVI 5.5 (Harris Geospatial, Boulder, California), and R.

### Site selection and randomization

**Fig 2:**
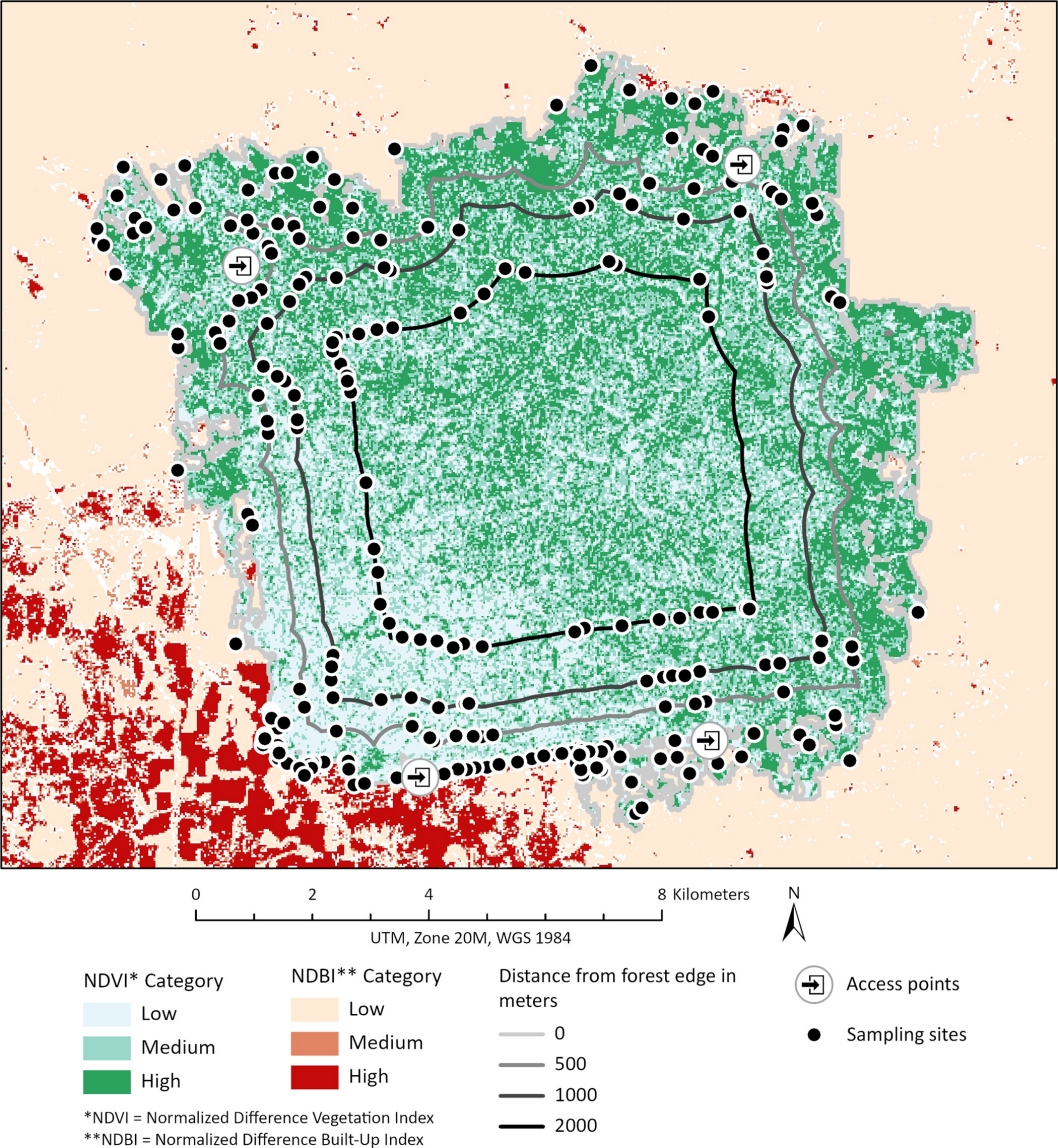
Map of the Ducke reserve, surrounding areas, and sampling sites. The map shows the Ducke reserve and contiguous forest categorized by Normalized Difference Vegetation Index (NDVI) at a 30 m spatial resolution, while the surrounding areas are categorized by Normalized Difference Built-up Index (NDBI) at the same resolution. Both layers were derived from Landsat 8 Operational Land Imager surface reflectance imagery obtained from the USGS Earth Explorer data portal [24]. Sites sampled (black dots) are shown for each distance (continuous lines). The main access points are in the north and south of the reserve.

Buffer distances were drawn at the forest edge (0 m), and 500, 1000, and 2000 m into the forest. Initially, nine potential sampling sites were randomly generated within each distance and NDVI category (= 108 sites). A further 293 potential sampling sites were generated using the same methods, as required. In addition, 179 potential sampling sites were randomly generated along the southwestern edge of the Ducke reserve bordering the city to intensify sampling in proximity to humans. The latter sites were not stratified by NDVI category, although NDVI values were extracted from all sampling sites for analysis. On a given day, forest edge and forest interior sites were either sampled simultaneously, or sampling alternated daily between the edge and interior, depending on logistics of accessing sites. Interior sites were selected from those available within approximately 5 km of an access point (Fig 2). In total, 244 of the 587 potential sites were sampled over the course of the study, some of which were sampled twice. Of the unique sites, 196 were stratified by distance from forest edge and NDVI category while the remainder were randomly generated along the southwestern edge of the reserve.

### Mosquito collections

Mosquitoes were usually sampled twice per week across two rainy seasons from January to June 2019, and November 2019 to April 2020, using the following mechanized and human-operated methods (Table 1): BG-Sentinel 2 traps (BioQuip, Rancho Dominguez, California) baited with dry ice and BG-Lure [15], 20 cm diameter hand-nets, and Prokopack aspirators (John W. Hock Company, Gainesville, Florida). BG-Sentinel traps and aspirators were used at sampling sites for the entire study while hand-net collections were initiated one month after the start of the study. Each sampling site was located using a Garmin Rino 755t (Garmin Ltd., Olathe, Kansas) handheld GPS. BG-Sentinel traps were prepared as described previously [13,18] and left to run until the next day. If traps failed (usually due to battery failure), data were excluded from the BG-Sentinel trap dataset (S1 Dataset). Hand-nets were used to manually sample all approaching mosquitoes for approximately 10 minutes while standing within 5 m of traps. Aspirators were used to sample mosquitoes from all vegetation up to chest height within a 5 m radius of traps. We generally sampled with hand-nets on the day traps were placed and with aspirators on the day traps were collected. BG-Sentinel traps were used regardless of weather. Heavy rainfall occasionally limited the use of hand-nets and aspirators, although we were usually able to utilize these on one of the two days associated with each trap collection. Mosquitoes were stored on dry ice at the time of collection and were transferred to a -80°C freezer at the Fundação de Medicina Tropical Doutor Heitor Vieira Dourado (FMT-HVD) until identified.

**Table 1:** Properties of BG-Sentinel traps [15], hand-nets, and Prokopack aspirators [14] influencing their attractiveness to mosquitoes and usefulness as sampling methods.

	BG-Sentinel	Hand-net	Prokopack aspirator
Primary use	Collection of urban *Aedes* and *Culex* species mosquitoes [15]	Collection of anthropophilic mosquitoes [16]	Indoor sampling of resting mosquitoes [14]
Mechanized component	12V battery-powered electrical fan	None	12V battery-powered electrical fan
Human-operated (Yes/No)	No (although possible residual effect of handling traps) [28]	Yes	Yes
Visual cues	Trap body composed of dark-blue material	Natural (human operator)	Natural (human operator)
Olfactory cues	BG-Lure (ammonia, lactic acid, and caproic acid), CO_2_ (dry ice)	Natural (human operator)	Natural (human operator)
Thermal cues	Mimics convection currents created by human body	Natural (human operator)	Natural (human operator)
Specimen quality	Susceptible to damage by electrical fan	High quality, ideal for morphological identification	Susceptible to damage by electrical fan

### In situ environmental variables

In situ environmental variables were recorded at each site to investigate associations with distance from edge and the occurrence (presence/absence) of mosquito species. Temperature (°C) and relative humidity (%) were recorded at 30-min intervals for the duration of each BG-Sentinel trap collection using Hygrochron iButton data loggers (Maxim Integrated, San Jose, California) placed in a nylon mesh bag (BioQuip, Rancho Dominguez, California) and attached to the outside of each trap. The minimum, maximum, mean, daytime mean (06:00–18:00), and range of both temperature and relative humidity were calculated for BG-Sentinel trap collections. Values for hand-nets and aspirators were extracted from the datapoint nearest to the time of the collection. Elevation (m) values for each sampling site were calculated as previously described [13]. The % canopy cover was recorded using a spherical densiometer (Forest Densiometers, Rapid City, South Dakota) based on readings made directly above the trap and 5 m from the trap in N, SE, and SW directions. The number of palms with open fronds and the number of mature trees within a 5 m radius of the trap was recorded. Mature trees were defined as those other than palms with a diameter at the base of the trunk of approximately 30 cm or more. The % ground vegetation cover (ground cover) below chest height was estimated in 1 m^2^ transects at distances 5 m from the trap in N, S, E, and W directions, and expressed as a value: 1 = 0–25%, 2 = 25–50%, 3 = 50–75%, and 4 = 75–100% total cover. Average ground cover was then calculated as the sum of these values, divided by four. Trap substrate was recorded as the estimated % leaf litter, % soil, % grass, % palm frond, % sand, and % other vegetation, present directly beneath the trap, with the total of each type combined to equal 100%. A measure of the slope (°) representative of the general area where the trap was placed was made using the Rotating Sphere Inclinometer v1.8 smartphone app (Calmatics, Gothenburg, Sweden) and the aspect of the slope (°) was recorded using a Garmin Rino 755t handheld GPS.

### Mosquito identification

Mosquitoes were placed on a chill table (BioQuip, Rancho Dominguez, California, USA) and identified to species using a stereomicroscope and taxonomic keys as previously described [18]. Genus and species names follow Wilkerson et al. [29]. Samples were stored at -80°C for future arbovirus screening.

### Statistical analysis

Contingency tables and two-tailed Fisher’s exact tests were used to compare the genus-level occurrence of *Haemagogus* and *Sabethes* mosquitoes across sampling methods between forest edge and interior sites. For this comparison, a single sample bout (i.e., where all sampling methods were employed within ≤14 days) was randomly downselected from each site that was sampled twice, and a very small number of sites from which both genera were collected were excluded. To analyze patterns of collection across the two rainy seasons, data were grouped into three-month sampling blocks using the BG-Sentinel trap dataset (1 = January–March 2019; 2 = April–June 2019; 3 = November 2019 –January 2020; 4 = February–April 2020). Data were grouped this way to generate replicates for analysis while maintaining an acceptably balanced sample size among groups. Mann-Whitney U tests followed by Wilcoxon Each Pair tests were used to compare genus-level abundance across sampling blocks. Two-tailed Fisher’s exact tests were used to compare species-level occurrence across sampling blocks. For the latter, species with excess zero counts were excluded. For both comparisons, a Bonferroni correction was used to adjust alpha for multiple tests.

Analyses of community similarity, richness, and diversity were only performed using samples identified to species level. To compare mosquito communities across distances into the forest, a principal components analysis of relative frequencies of each species was conducted, followed by hierarchical clustering of communities at each distance, as in our previous studies [17,18]. The Morisita overlap index was also used to compare community composition at each distance using the PAST software package [30]. To examine whether sampling was adequate to capture total species richness, iNEXT [31] (R version 4.2.1) was used to generate species accumulation and rarefaction curves by distance for each sampling method as well as for all methods combined (223 sites where all three methods were used on the same sampling occasion). The Shannon-Wiener diversity index was calculated using data grouped in three-month blocks as described above and ANOVA, followed by a Tukey HSD post-hoc test, was used to detect differences in diversity between distances for each sampling method as well as for all methods combined.

The BG-Sentinel trap dataset was used to measure associations of environmental variables with each other and with distance into forest, as this was the largest of the assembled datasets. A single sample was randomly downselected from each site sampled twice, resulting in 235 unique sites for analysis. A Spearman’s rank correlation was then used to test associations between environmental variables, of which six were selected for further analysis based on the generated matrix and our previous work [13]. These were: elevation, maximum temperature, NDVI value, average ground cover, number of palms, and number of mature trees. To compare these variables among the four distances sampled, ANOVA was used when data were normally distributed followed by a Tukey HSD post-hoc test to detect pairwise differences, while the Kruskal-Wallis test was used for non-normal data followed by a Steel-Dwass all-pairs comparison to detect pairwise differences.

Contingency tables and two-tailed Fisher’s exact tests were used to test species occurrence across sites sampled twice for sufficiently common taxa (*Aedes*, *Ae*. *albopictus*, *Culex*, *Limatus*, *Li*. *durhamii*, *Ps*. *amazonica*, *Sabethes*, *Trichoprosopon*, and *Wyeomyia*), to determine whether these sites could be treated as independent samples or needed to be nested. All sample data for each trapping method were then used in nominal logistic regressions to test associations between distance from forest edge (as a categorical variable) and six selected, continuous environmental variables with the occurrence of key taxa. Variables were removed sequentially from the model until all remaining variables contributed significantly or only one variable was left. Analyses of the BG-Sentinel trap dataset were also repeated using only single samples per site, which were randomly downselected as described above.

Since the study aimed to better understand the potential exchange of pathogens at ecosystem boundaries, a Mann-Whitney U test was used to test the association of NDBI values in a 100 m radius around the sampling site with the occurrence of key vector taxa. This radius was chosen for its relevance to average ranges of mosquito movement, but it was significantly correlated with all other NDBI variables. A single sample bout was downselected from sites sampled twice and detection was combined for all three sampling methods, resulting in a total of 100 sites for analysis.

Statistical analyses were performed with JMP Pro 15 [32] unless otherwise stated.

## Results

### Relative mosquito abundance and general patterns of collection

**Fig 3:**
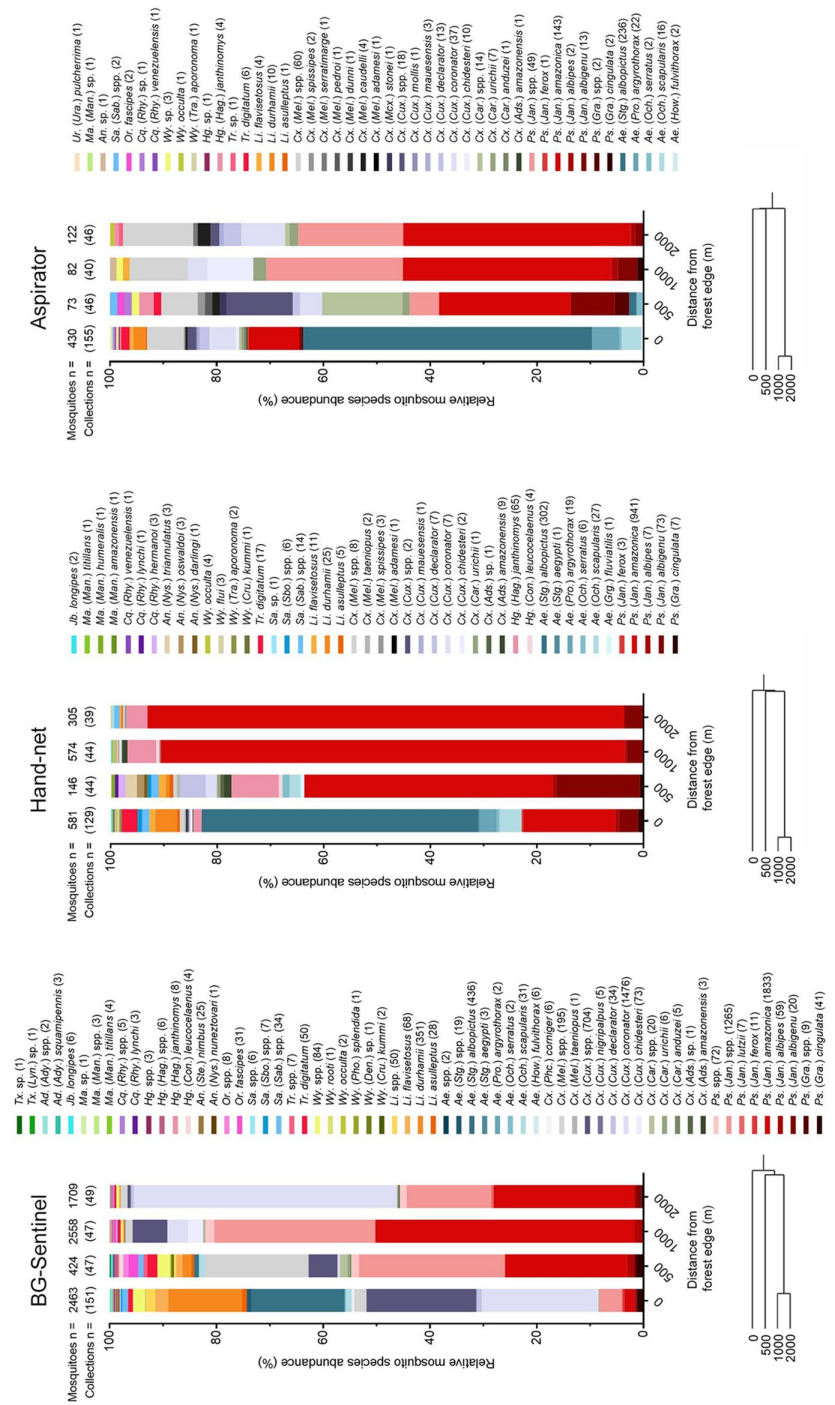
Relative mosquito species abundance by distance from forest edge for BG-Sentinel traps, hand-nets, and aspirators. Stacks ordered by genus abundance and then alphabetically by subgenus and species. Number of mosquitoes (Mosquitoes n =) and number of collections (Collections n =) made at each distance shown above bar. Number of individuals per taxon included in parentheses next to corresponding name; sp. = single species, spp. = potentially multiple species. Abbreviated names are given in full in the dataset (S1 Dataset). Dendrograms below each graph show hierarchical clustering of principal component (PC)1 and PC2 from a principal components analysis of relative species frequency for each collection method at each distance sampled.

A total of 9,467 mosquitoes were sampled across 244 unique sites, of which several were sampled twice (detailed below), using BG-Sentinel traps, hand-nets, and Prokopack aspirators (S1 Dataset). The relative abundance and composition of mosquitoes varied by sampling method and distance from the forest edge (Fig 3). All specimens were identified to the rank of genus, and often subgenus, but poor sample condition sometimes prevented species-level identification. Specimens collected with hand-nets were in excellent condition and 97.8% were identified to species level, while those collected with aspirators (80.6% identified to species level) and BG-Sentinel traps (65.0%) were more likely to be damaged. Overall, collections yielded 16 genera and 57 identified species. BG-Sentinel traps (N = 294 sites sampled in total, including 59 sites sampled twice) yielded the most mosquitoes (7,154), comprising 15 genera and 38 identified species, of which 94.4% were female; 1,606 mosquitoes were sampled with hand-nets (N = 256 sites, including 24 sites sampled twice, 12 genera, 40 species, 89.7% female), and 707 mosquitoes were sampled with Prokopack aspirators (N = 287 sites, including 51 sites sampled twice, 13 genera, 35 species, 65.1% female).

*Psorophora* was the most abundant genus sampled with BG-Sentinel traps and hand-nets, while *Aedes* was the most abundant genus sampled with aspirators (Fig 3). *Psorophora amazonica* and *Ae*. *albopictus* were the dominant species within respective genera and both were sampled in high relative abundance using each method. Both *Psorophora* generally and *Ps*. *amazonica* specifically were present in higher relative abundance in the forest interior than at the edge, while *Ae*. *albopictus* was predominantly found at the forest edge, occasionally at 500 m, and rarely at 1000 m into the forest. *Culex* species were present in high relative abundance in BG-Sentinel traps and aspirators but were unlikely to be biting humans in high numbers during the daytime since they were seldom collected in hand-nets. *Sabethes* species were more common in BG-Sentinel traps than hand-nets while the opposite was true for *Haemagogus* generally and *Hg*. *janthinomys* specifically. Neither *Sabethes* nor *Haemagogus* were common in aspirator collections. Contingency table analyses of species presence at each site revealed that *Sabethes* mosquitoes (all species combined) were more common than *Haemagogus* mosquitoes (all species combined) at the edge and less common in interior sites when either net collections (Fisher’s exact test, DF = 2, χ^2^ = 15.5, P = 0.0004) or BG-Sentinel trap collections (DF = 2, χ^2^ = 13.0, P = 0.002) were analyzed; too few specimens were collected using aspirators to permit analysis.

Differences in genus-level abundance across three-month sampling blocks were only significant after a Bonferroni correction for multiple testing for *Culex* (Mann-Whitney U test, DF = 3, χ^2^ = 13.8, P = 0.003), which was most abundant in blocks 1 and 4, and *Trichoprosopon* (DF = 3, χ^2^ = 21.9, P < 0.0001), which was most abundant in blocks 1 and 2. Comparisons of species occurrence showed significant differences for *Li*. *durhamii* (DF = 3, χ^2^ = 14.7, P = 0.002), *Ps*. *amazonica* (DF = 3, χ^2^ = 15.6, P = 0.001), and *Tr*. *digitatum* (DF = 3, χ^2^ = 24.1, P < 0.0001), all of which were more common in month blocks 1 and 2 than in blocks 3 and 4, corresponding with higher occurrence in the first rainy season. There was no difference in the occurrence of *Ae*. *albopictus* among sampling blocks (P > 0.05).

### Analyses of community similarity, richness, and diversity

In a principal components analysis of the relative frequency of each species at each distance, the first two principal components (PC1 and PC2) explained 89.2% of the variation in the BG-Sentinel trap data, 92.6% of variation in hand-net data, and 80.9% of variation in aspirator data. For BG-Sentinel trap data, PC1 reflected the relative abundance of a group of 17, predominantly rare species, though this group did include *Li*. *durhamii* and *Ae*. *albopictus*, while PC 2 reflected the relative abundance of six additional, rare species. Generally, the loadings were similar for the aspirator data, albeit PC1 also reflected the relative abundance of *Ps*. *amazonica*. However, loadings were strikingly different for the hand-net data, where PC1 reflected the relative abundance of *Hg*. *janthinomys*, while PC2 reflected the abundance of four rare species that did not, with one exception (*Ps*. *cingulata* in BG-Sentinel trap data), load heavily onto PC1 or PC2 in the other two datasets. Hierarchical cluster analysis of PC1 and PC2 (Fig 3) revealed that, for all sampling methods, communities at 1000 m and 2000 m were quite similar but differed substantially from communities at 500 m, which in turn differed from communities at the edge.

The Morisita overlap index (S1 Table) derived from hand-net and aspirator collections showed that forest edge communities differed from those sampled at 500, 1000, and 2000 m (Morisita index < 0.32 for all comparisons), while interior distances were quite similar when compared with each other (> 0.80 for all comparisons). When this index was calculated using BG-Sentinel trap data, communities at 500 m and 1000 m were more distinct from edge communities than those at 2000 m. However, this disparity was due to large numbers of *Cx*. *coronator* collected in traps at 2000 m over two days in January 2019 (99.8% of the total *Cx*. *coronator* collected at this distance). If removed, the results followed a similar trend to both hand-nets and aspirators.

**Fig 4:**
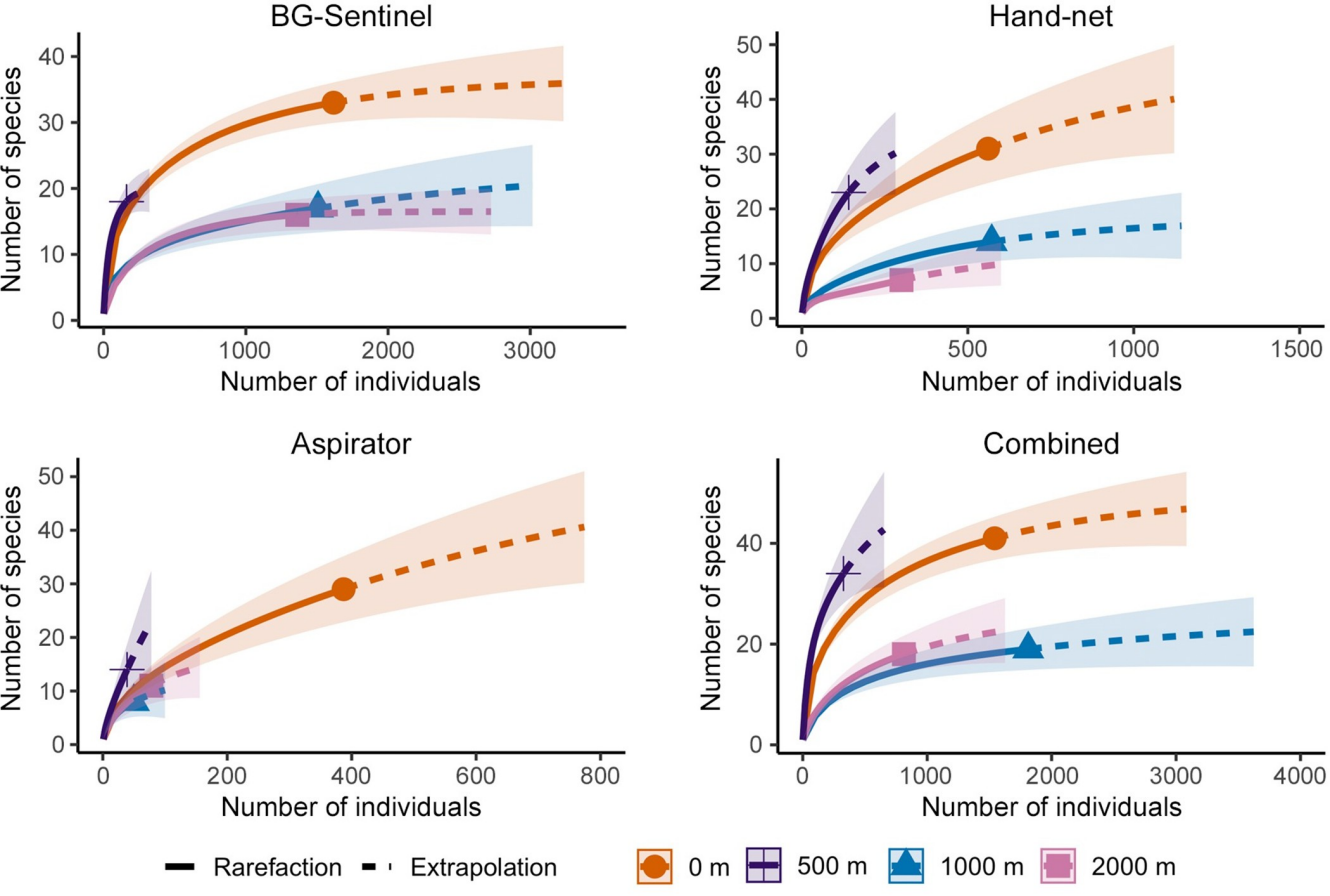
Species accumulation and rarefaction curves. Panels show species richness by distance for each sampling method, and combined data for 223 sites where all three methods were used on the same sampling occasion. Shaded areas surrounding rarefaction and extrapolation lines represent 95% confidence intervals. Curves generated using taxa identified to species level only.

Species accumulation and rarefaction curves showed that species richness was generally higher at 0 m and 500 m than at 1000 m and 2000 m across the three sampling methods and for all methods combined (Fig 4). Results were more difficult to interpret for Prokopack aspirators, which were relatively ineffective inside the forest where fewer sites were sampled at each distance compared to forest edges. Overall, species were well represented at 0, 1000, and 2000 m, although some rarer species remained to be sampled. The steep increase in accumulation curves at 500 m shows that additional sampling was needed to capture the total richness at this distance.

Species diversity, as measured by the Shannon-Wiener diversity index, differed significantly across distance from forest edge when sampling with hand-nets (ANOVA, DF = 3, F = 8.06, P = 0.003) and for all methods combined (ANOVA, DF = 3, F = 6.83, P = 0.006). For hand nets, these differences were significant between 0 m and 1000 m, 0 m and 2000 m, and 500 m and 2000 m (Tukey HSD, P < 0.05 for all comparisons), and for all methods combined, differences were significant between 0 m and 1000 m, 0 m and 2000 m, and 500 m and 1000 m (Tukey HSD, P < 0.05 for all comparisons). Diversity was generally higher at 0 m and 500 m than at 1000 m and 2000 m (S2 Table). Differences in diversity were not significant when sampling with BG-Sentinel traps (ANOVA, DF = 3, F = 1.94, P = 0.18) or aspirators (ANOVA, DF = 3, F = 2.30, P = 0.13), although both showed similar trends for higher diversity towards the forest edge.

### Differences in environmental variables by distance into forest

**Fig 5:**
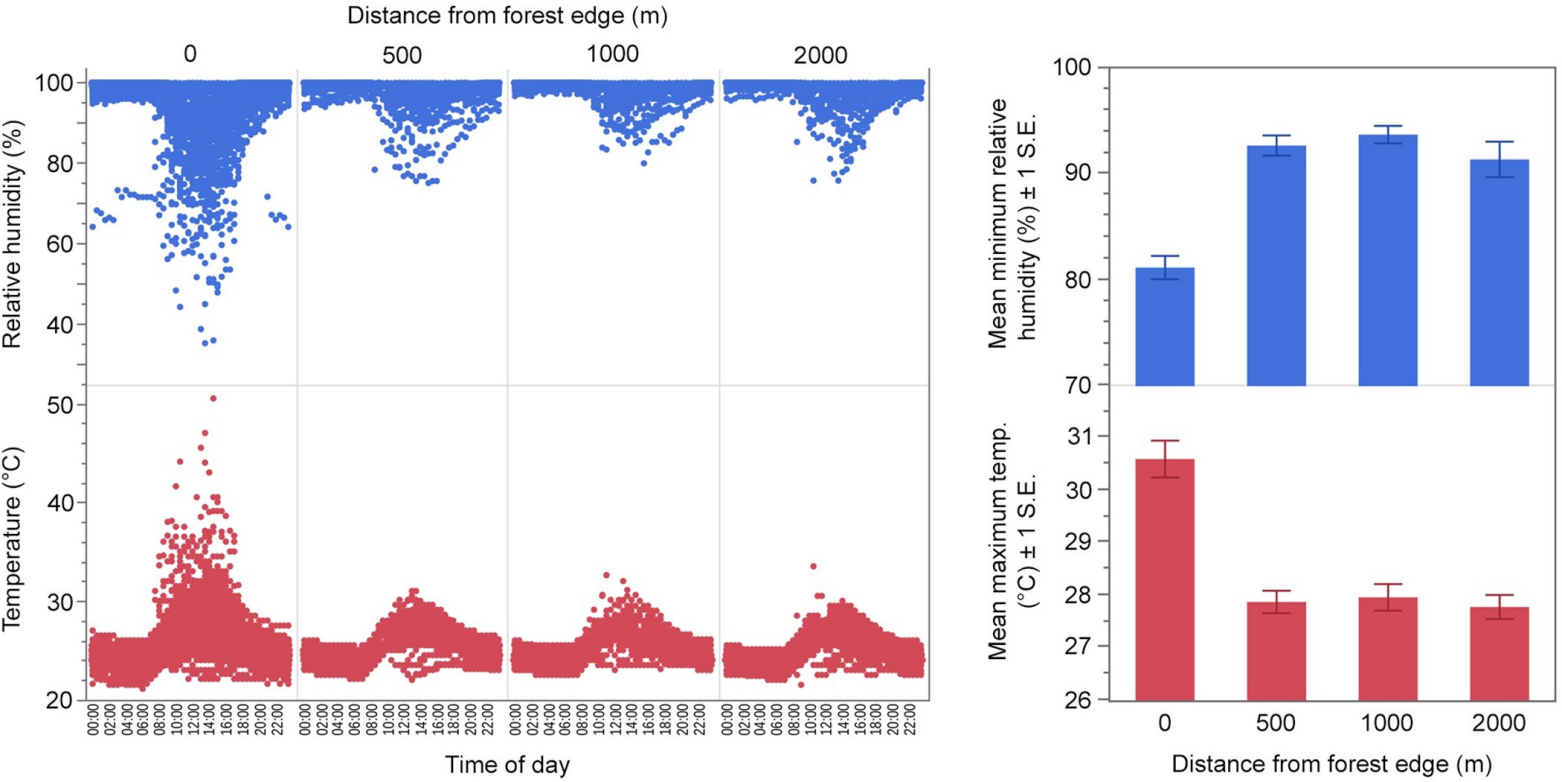
Variation in microclimate. Left panel shows daily cycles of temperature (°C) and relative humidity (%) recorded at 30-min intervals for each distance from forest edge. Data points derived from all BG-Sentinel trap collections where temperature and relative humidity were recorded, including those where traps failed (S1 Dataset). Right panel shows mean minimum relative humidity (%) and mean maximum temperature (°C) ± 1 standard error (S.E.) at each distance sampled, constructed using data from the final BG-Sentinel trap dataset where traps were still working upon collection.

A Spearman’s rank correlation showed that measures of NDVI and NDBI were generally inversely correlated, measures of microclimate were highly correlated, and most of the environmental variables measured, except for elevation, were correlated to some degree (S3 Table). Of the six environmental variables selected for further analysis (elevation, maximum temperature, NDVI value, average ground cover, number of palms, and number of mature trees), elevation increased slightly (15 m) but significantly (ANOVA, DF = 3, F = 5.85, P = 0.0007) between 0 m and 500 m into the forest; there was no significant difference in elevation between sites at 500, 1000, and 2000 m (S4 Table). NDVI followed a similar trend, increasing significantly (Kruskal-Wallis, DF = 3, χ^2^ = 60.7, P < 0.0001) between 0 m and 500 m and remaining steady thereafter. Forest edge sites were hotter and less humid than each of the interior distance categories (Fig 5). Maximum temperature decreased significantly between 0 m and 500 m (Kruskal-Wallis test, DF = 3, χ^2^ = 29.3, P < 0.0001) and remained stable at 500, 1000, and 2000 m. Average ground cover decreased continuously from 0 m to 1000 m and subsequently leveled off (Kruskal-Wallis, DF = 3, χ^2^ = 30.3, P < 0.0001). The number of palms and mature trees increased abruptly between 0 m and 500 m and leveled off across the remaining distance categories (Kruskal-Wallis, DF = 3, χ^2^ = 96.1 for palms and 59.3 for mature trees, P < 0.0001 for both comparisons).

### Associations between select variables and the occurrence of key taxa

Among the common taxa sampled with BG-Sentinel traps (*Aedes*, *Ae*. *albopictus*, *Culex*, *Limatus*, *Li*. *durhamii*, *Ps*. *amazonica*, *Sabethes*, *Trichoprosopon*, and *Wyeomyia*), only *Limatus* showed a significant association (P = 0.008) between the first and second sample at the same site after a Bonferroni correction. This comparison remained significant when limited to just the 0 m distance. No associations were significant for hand-net collections, and aspirators generally collected too few samples for analysis. The average time between the first and second sample was 84 days (min. = 40 d, max. = 143 d).

Nominal logistic regressions used to test associations between the six selected environmental variables and the occurrence of key taxa showed that three taxa were associated with forest edges (Table 2). Of these, *Ae*. *albopictus* showed strong negative associations with distance from forest edge and positive associations with elevation across all sampling methods, in addition to negative associations with NDVI value and the number of mature trees across one of the three sampling methods. *Aedes scapularis* showed negative associations with distance from forest edge, temperature, and the number of mature trees, and positive associations with elevation and NDVI value across at least one of the three sampling methods. *Limatus durhamii* showed strong negative associations with distance from forest edge and positive associations with NDVI value and average ground cover, although the latter was only marginally significant. Of the forest specialists, *Ps*. *amazonica* showed positive associations with distance from forest edge, elevation, NDVI value, and the number of mature trees, and a negative association with temperature. However, each of these associations was only significant for one of the three sampling methods, despite *Ps*. *amazonica* being the most frequently encountered species. *Haemagogus* generally and *Hg*. *janthinomys* specifically showed positive associations with distance from forest edge. In contrast, there was no association between distance from forest edge and the occurrence of either subgenus of *Sabethes* mosquitoes, although *Sabethes* (*Sabethes*) spp. showed positive associations with temperature and average ground cover.

**Table 2:** Results from nominal logistic regressions (P value and (χ^2^)) to test associations between key variables on occurrence of mosquito taxa captured with BG-Sentinel traps (BGS, N = 294 sites), hand-nets (NET, N = 256 sites), and backpack aspirators (ASP, N = 287 sites). Blue and red shaded cells indicate significant positive or negative associations, respectively. Gray shaded cells represent factors that were not significantly associated with the designated taxon.

		Variables included in model						
Species/ Taxon	Sampling method (N sites positive)	Distance from forest edge	Elevation	Temperature (Max for BGS; actual for NET and ASP)	NDVI value	Average ground cover	Number palms	Number mature trees
*Ae*. *albopictus*	BGS (61)	<0.0001* (78.0)	0.04 (4.4)					0.02* (5.5)
	NET (66)	<0.0001 (76.1)	0.002 (9.5)		0.02 (5.7)			
	ASP (39)	<0.0001 (53.8)	0.004 (8.4)					
*Ae*. *scapularis*	BGS (8)			0.009* (6.9)				0.02† (5.7)
	NET (15)	0.003 (14.2)	0.02 (5.4)					
	ASP (8)	0.002 (15.2)			0.04 (4.2)			
*Haemagogus* (*Hag*.) spp.^ǂ^	BGS (10)	0.043* (8.1)						
*Hg*. *janthinomys*	BGS (5)							
	NET (24)	0.047 (7.9)						
*Li*. *durhamii*	BGS (36)	<0.0001* (29.7)			0.002* (9.7)			
	NET (20)	0.002 (15.3)				0.048 (3.9)		
	ASP (6)	0.03 (9.0)						
*Ps*. *amazonica*	BGS (48)		0.009* (6.8)		0.0001* (14.8)			
	NET (110)	<0.0001 (21.5)		0.001 (10.3)				0.005 (8.0)
	ASP (42)							
*Sabethes* (*Sab*.) spp.^ψ^	BGS (17)			0.001* (10.0)		0.009† (6.8)		
*Sabethes* (*Sbo*.) spp.^ψ^	BGS (6)		0.04* (4.1)				0.04* (4.25)	

*Comparison remained significant when one of two sampling events was selected randomly and excluded, resulting in N = 235 unique sites. This downselection was only conducted for BG-Sentinel trap data.

†Comparison approached significance (P < 0.08) when one of two sampling events was selected randomly and excluded, resulting in N = 235 unique sites. This downselection was only conducted for BG-Sentinel trap data.

^ǂ^Analyses performed at species level (*Hg*. *janthinomys*) in hand-nets.

^ψ^Insufficient numbers for analyses in nets and aspirators.

### Associations between surrounding levels of NDBI and the occurrence of key taxa

Sites in which *Ae*. *aegypti* (Mann-Whitney U test, N = 3 positive sites, DF = 1, χ^2^ = 6.2, P = 0.01) and *Ae*. *albopictus* (N = 65 positive sites, DF = 1, χ^2^ = 19.7, P < 0.0001) were detected had significantly higher mean NDBI values in a 100 m radius around the site than sites where these species were not detected. *Sabethes* mosquitoes showed an opposite trend, with positive sites (N = 22) having lower levels of NDBI within 100 m, albeit the difference was only marginally significant (χ^2^ = 3.7; P = 0.06). However, sites in which *Haemagogus* (N = 6) or *Psorophora* mosquitoes (N = 50) were detected did not differ in mean NDBI value of the surrounding area when compared to sites where they were not detected (P > 0.48 for both comparisons). S5 Table shows the mean distance to low, medium, and high NDBI pixels for sites in which select taxa were detected.

## Discussion

This study expands upon our earlier investigation of ground-level changes in mosquito community composition at the edges of urban forest fragments in Manaus, where we aimed to identify potential routes of spillover and spillback of mosquito-borne viruses [13]. We previously focused on relatively small (≤6.6 km^2^) forest fragments embedded within the urban matrix, bordering densely populated areas, where humans, mosquitoes, and wildlife could readily interact. In contrast, the Ducke reserve covers 100 km^2^ of primary rainforest [19] and while its southwestern edge forms a striking interface with the city, the remaining edges border rural areas which are more or less contiguous with the surrounding Amazon rainforest.

We used three complimentary sampling methods to maximize representation of mosquito fauna in the study area. Species abundance was highest among BG-Sentinel trap collections (75.6% of all mosquitoes sampled), which was expected and agrees with other work in forests utilizing multiple sampling methods [16,33]. However, these data were somewhat inflated by relatively rare sampling events of large numbers of *Psorophora* and *Culex* mosquitoes (S1 Dataset). The overall richness of identified species was similar across the three sampling methods. Species richness was similarly high at the forest edge among BG-Sentinel trap, hand-net, and aspirator collections, and was higher towards the edge (0 m and 500 m) for all methods combined. Species richness was generally lower at interior sites (1000 m and 2000 m), particularly among hand-net collections, where fewer anthropophilic species were present and *Ps*. *amazonica* dominated. Aspirators were relatively ineffective when sampling mosquitoes inside the forest, likely due to the wealth of available resting sites, particularly above the ground.

When considering key vectors, each sampling method showed *Ae*. *albopictus* to be present in high relative abundance at forest edges. This species was also present 500 m and 1000 m into the forest, but only in very low numbers, agreeing with studies conducted at the same reserve [34] and in the Tijuca Forest, Rio de Janeiro [35]. We rarely encountered *Ae*. *aegypti*, which was only sampled at three sites along the urban edge. *Haemagogus* and *Sabethes* mosquitoes formed less than 2% of the overall catch which was expected since these genera comprise mostly canopy-dwelling species [18]. Whereas *Haemagogus* mosquitoes readily approach humans [36], *Sabethes* approach more slowly and are easily disturbed by movement [37], a behavior we have repeatedly observed in the field. Consequently, short duration hand-net collections, which were effective at detecting *Hg*. *janthinomys*, seemed less effective for sampling *Sabethes*. In contrast, longer duration collections with dry-ice-baited BG-Sentinel traps seemed better for this purpose.

The vector status of *Ps*. *amazonica* is currently unknown. However, it is a highly seasonal, highly anthropophilic species, which is mainly present during the latter months of the rainy season and has been well represented in each of our previous studies [13,17,18]. In the present study, *Ps*. *amazonica* formed 92.2% of the identified *Psorophora*, and was particularly abundant in hand-net collections, reinforcing its status as an aggressive human biter. Crucially, its substantial presence at each distance and height [17] sampled provides a clear potential pathway for pathogen exchange between the forest edge and canopy. Vector competence studies involving this species are clearly needed.

We found differences in the abundance and occurrence of certain taxa across three-month sampling blocks and between rainy seasons. *Trichoprosopon* was the only genus with higher abundance in month blocks 1 and 2, corresponding to the first rainy season, compared to month blocks 3 and 4. The occurrence of *Li*. *durhamii*, *Ps*. *amazonica*, and *Tr*. *digitatum* was also significantly higher during month blocks 1 and 2. We previously observed peaks in the abundance and occurrence of *Limatus*, *Psorophora*, and *Trichoprosopon* during April and May when sampling smaller forest fragments in Manaus [13]. Since our sampling was truncated midway through the second rainy season by the COVID-19 pandemic, we likely missed periods of peak activity for some of these taxa.

Overall, forest edges differed in richness and diversity of mosquito communities compared to the forest interior, with communities at 1000 m and 2000 m being most alike and least diverse. Some groups of organisms are thought to be diverse and species rich at forest edges where interior and exterior species coexist along with edge specialists [11,38,39]. In this regard, mosquitoes have not been extensively studied and results of field investigations are inconclusive. Steiger at al. [11,12] reported contrasting findings when analyzing species richness across grassland, edge, and interior habitats of an Australian tropical forest, but noted significant changes in community composition between the edge and interior in both studies. We found no difference in species richness and diversity when sampling up to 500 m from the forest edge in our previous study [13], but like Steiger et al. [11,12], we detected substantial changes in community composition. Young et al. [40] conducted a more intricate study of mosquito communities at forest edges in Malaysian Borneo, finding little difference in richness, diversity, and community composition when sampling interior distances up to 500 m from the edge. We sampled deeper into the forest and at a coarser resolution in the present study, where we generally found a peak in richness and diversity towards the edge, which declined beyond 500 m. Species accumulation curves showed that rarer taxa remained to be sampled, particularly at 500 m, although these are less likely to serve as bridge vectors given their relative scarcity. Similar results from other studies highlight the difficulty of sampling mosquito species to saturation at ground level in forested environments [40,41], especially given biases associated with different sampling methods [16,33,42].

Analyses of the six selected environmental variables showed that changes mainly occurred between the forest edge and 500 m. Thereafter, conditions remained relatively stable, apart from average ground cover which gradually declined up to 1000 m. These findings are in broad agreement with the permeability of forest edges to external biotic and abiotic factors elsewhere near Manaus [39]. We found that NDVI value was lowest at forest edges and increased significantly by 500 m, agreeing with our earlier study [13] and others who have shown similar changes within 150 m of urban-forest edges [43]. Field observations have shown that desiccating effects can penetrate up to 200 m into forests [39], altering microclimate by creating hotter and drier conditions [13]. Satellite observations have shown that these effects can penetrate beyond 1 km in moderately and highly fragmented forest [44]. Tree mortality is often elevated at forest edges, disproportionally affecting larger trees [45] and adult palms [46], which may lead to increased light penetration and successional plant growth [47]. In accordance, we detected an increase in the number of mature trees and palms, and a decrease in average ground cover moving from the forest edge to interior. We also detected a slight (15 m) increase in elevation between the edge and 500 m. While changes in topography may influence abiotic conditions and vegetation characteristics [48], the effects are likely to be minimal given the small difference we encountered. Many of the above findings agree with the results of our previous study in urban forest fragments in Manaus [13].

Analyzing associations between distance from forest edge and the six specified environmental variables with species occurrence highlighted several traits associated with key taxa. *Aedes albopictus* was negatively associated with distance from forest edge across all sampling methods, as well as with NDVI value and the number of mature trees when sampled with nets and traps, respectively, agreeing with its preference for edge habitats among forested areas [13,34]. Although generally considered a peri-urban species which occasionally breeds in water-gathering tree holes [49], dense primary vegetation may not represent ideal habitat for *Ae*. *albopictus*. Other species associated with forest edges included *Ae*. *scapularis* and *Li*. *durhamii*, which were mainly found within 500 m of the border. The former showed similar trends to *Ae*. *albopictus* but was negatively associated with temperature and positively associated with NDVI value across one of the three sampling methods, suggesting it may be better adapted to areas of more dense vegetation. Indeed, *Ae*. *scapularis* is commonly found in forests [50,51] but is also well adapted to human environments [52] and has potential to transmit multiple viruses of public health importance including YFV [53,54] and MAYV [55]. *Limatus durhamii* showed strong negative associations with distance from forest edge and positive associations with NDVI value and average ground cover, although the association with the latter was only weak. This species breeds in both natural and artificial containers [56] and adult females are attracted to humans [51], but there is currently only limited evidence of a role for *Limatus durhamii* in arbovirus transmission [57].

*Psorophora amazonica* showed positive associations with several variables suggestive of forest-dwelling species (distance from forest edge, NDVI value, and the number of mature trees), but these associations were not consistent across sampling methods. This was true for most variables, other than distance from forest edge, associated with the key taxa listed in Table 2. We detected a negative association between *Ps*. *amazonica* and temperature when sampling with hand-nets. We previously found a negative association with temperature when sampling *Ps*. *albigenu*, but not *Ps*. *amazonica*. It seems that *Psorophora* (*Janthinosoma*) species are generally more abundant during cooler conditions (< 26°C) than their forest-dwelling counterparts, although they tolerate a wide range of temperatures [17]. Of the variables analyzed, *Haemagogus* generally and *Hg*. *janthinomys* specifically showed positive associations with distance from forest edge. These taxa were sampled in relatively low numbers at ground level, which may be why we failed to detect associations with temperature and humidity that we recorded previously when sampling vertically [17,18]. Since *Hg*. *janthinomys* breeds mainly in tree holes in primary tropical rainforest [36,58], the dramatic environmental shifts occurring between 0 m and 500 m, where large tree mortality is elevated [45], may explain its relative scarcity compared to *Sabethes* species mosquitoes. The latter, which breed among a range of phytotelmata including bamboo internodes [59,60], may be better suited to forest edges. In support of this, we found no associations between the occurrence of *Sabethes* taxa and distance from forest edge at subgenus level, but we did find positive associations between *Sabethes* (*Sabethes*) spp., temperature, and average ground cover, which are higher at forest edges.

Zoonotic exchange of DENV, ZIKV, CHIKV, YFV, and MAYV requires spatiotemporal overlap of humans, susceptible monkeys (or other wildlife hosts), and competent mosquito vectors at or near forest edges. The proximity of human habitations to forest edges will influence the presence of non-native species in respective habitats (i.e., forest mosquitoes in human habitations and urban mosquitoes at forest edges). We used NDBI as a proxy for urbanization and found that sites positive for *Ae*. *aegypti* and *Ae*. *albopictus* had significantly higher mean NDBI values within a 100 m radius than sites that were not positive for these species. The mean distance of positive sites to the nearest medium NDBI pixel was similar for both species (S5 Table), while the mean distance to the nearest high NDBI pixel was greater for *Ae*. *albopictus*, in keeping with their respective preferences for urban and rural areas [61]. The greater mean distance of *Sabethes*, *Haemagogus*, and *Psorophora* positive sites to medium and high NDBI pixels suggests that these taxa are more likely to be encountered at forest edges bordering peri-urban or rural areas.

We sampled a forest reserve managed by the National Institute of Amazonian Research (INPA) where access is officially restricted. However, anecdotally, people do occasionally enter the reserve including for recreation, gathering fruit, and hunting. Illegal deforestation for the establishment of “invasion” communities, including the former Monte Horebe community on the western edge of the reserve [62], not only brings people into extremely close contact with recently cleared rainforest, but likely removes physical and social barriers associated with entering forests that exist in pre-planned urban areas. Behavioral studies are needed to better understand relationships between residents and forests bordering Manaus, and to better understand the risk of the spillover and spillback in these areas.

## Conclusion

Our findings suggest that major changes in environmental variables occur within 500 m of the forest edge at the Ducke reserve, where there is high risk for contact with both urban and sylvatic vectors, and that these are associated with substantial shifts in mosquito diversity and community composition. By 1000 m, conditions stabilize, species diversity decreases, and forest mosquitoes predominate. Environmental variables associated with the occurrence of key taxa may be leveraged to characterize suitable habitat and refine risk models for pathogen spillover and spillback.

## Supporting information

S1 Dataset Datasets for each sampling method and environmental variables. (XLSX)

S1 Table Morisita overlap index. (DOCX)

S2 Table Shannon-Wiener diversity index. (DOCX)

S3 Table Spearman’s rank analysis of environmental variables. (DOCX)

S4 Table Associations between select environmental variables and distance. (DOCX)

S5 Table Associations between NDBI and key taxa. (DOCX)
