# Supplementary material for: Where boundaries become bridges: Mosquito community composition, key vectors, and environmental associations at forest edges in the central Brazilian Amazon
Source: PLoS Negl Trop Dis. 2023 Apr 26;17(4):e0011296. doi: 10.1371/journal.pntd.0011296 (PMC10166490; doi:10.1371/journal.pntd.0011296)
Supplement: S1 Table — (DOCX) [file pntd.0011296.s002.docx]

**S1 Table.** Morisita overlap index by sampling method and distance for mosquitoes identified to species-level.

**BG-Sentinel** (all mosquitoes identified to species level)

|  | **0 m** | **500 m** | **1000 m** | **2000 m** |
| --- | --- | --- | --- | --- |
| **0 m** | 1 |  |  |  |
| **500 m** | 0.131 | 1 |  |  |
| **1000 m** | 0.099 | 0.940 | 1 |  |
| **2000 m** | 0.593 | 0.478 | 0.531 | 1 |

**BG-Sentinel** (with *Cx. coronator* collected at 2000 m removed)

|  | **0 m** | **500 m** | **1000 m** | **2000 m** |
| --- | --- | --- | --- | --- |
| **0 m** | 1 |  |  |  |
| **500 m** | 0.131 | 1 |  |  |
| **1000 m** | 0.099 | 0.940 | 1 |  |
| **2000 m** | 0.053 | 0.932 | 0.994 | 1 |

**Net**

|  | **0 m** | **500 m** | **1000 m** | **2000 m** |
| --- | --- | --- | --- | --- |
| **0 m** | 1 |  |  |  |
| **500 m** | 0.316 | 1 |  |  |
| **1000 m** | 0.285 | 0.822 | 1 |  |
| **2000 m** | 0.282 | 0.808 | 0.999 | 1 |

**Aspirator**

|  | **0 m** | **500 m** | **1000 m** | **2000 m** |
| --- | --- | --- | --- | --- |
| **0 m** | 1 |  |  |  |
| **500 m** | 0.220 | 1 |  |  |
| **1000 m** | 0.176 | 0.891 | 1 |  |
| **2000 m** | 0.184 | 0.897 | 0.965 | 1 |
