# Supplementary material for: Where boundaries become bridges: Mosquito community composition, key vectors, and environmental associations at forest edges in the central Brazilian Amazon
Source: PLoS Negl Trop Dis. 2023 Apr 26;17(4):e0011296. doi: 10.1371/journal.pntd.0011296 (PMC10166490; doi:10.1371/journal.pntd.0011296)
Supplement: S2 Table — (DOCX) [file pntd.0011296.s003.docx]

**S2 Table.** Mean Shannon-Wiener diversity index (SWI) ± 1 standard error (S.E.) at each distance for each sampling method and all methods combined using data grouped in three-month blocks: 1 = January – March 2019; 2 = April – June 2019; 3 = November 2019 – January 2020; 4 = February – April 2020. N = number of samples at each distance.

|  | **BG-Sentinel** | | **Hand-net** | | **Aspirator** | | **Combined** | |
| --- | --- | --- | --- | --- | --- | --- | --- | --- |
| **Distance** | N | Mean SWI  (± 1 S.E.) | N | Mean SWI  (± 1 S.E.) | N | Mean SWI  (± 1 S.E.) | N | Mean SWI  (± 1 S.E.) |
| **0 m** | 151 | 1.67 (0.13) | 129 | 1.51 (0.09) | 155 | 1.28 (0.21) | 112 | 1.83 (0.15) |
| **500 m** | 47 | 1.30 (0.44) | 44 | 1.43 (0.37) | 46 | 1.28 (0.22) | 42 | 1.70 (0.44) |
| **1000 m** | 47 | 0.82 (0.26) | 44 | 0.60 (0.18) | 40 | 0.59 (0.21) | 32 | 0.49 (0.12) |
| **2000 m** | 49 | 0.75 (0.33) | 39 | 0.27 (0.13) | 46 | 0.90 (0.24) | 37 | 0.67 (0.23) |
