# Supplementary material for: Where boundaries become bridges: Mosquito community composition, key vectors, and environmental associations at forest edges in the central Brazilian Amazon
Source: PLoS Negl Trop Dis. 2023 Apr 26;17(4):e0011296. doi: 10.1371/journal.pntd.0011296 (PMC10166490; doi:10.1371/journal.pntd.0011296)
Supplement: S3 Table — (DOCX) [file pntd.0011296.s004.docx]

**S3 Table.** Pairwise Spearman’s rank analysis of environmental variables, showing results of correlations. Blue cells = significant positive correlation; red cells = significant negative correlation; white cells show non-significant correlations. Spearman’s correlation coefficients (Rho) included in brackets.

|  | NDVI val | NDBI val | Dist low NDBI | Dist med NDBI | Dist high NDBI | NDBI max. | NDBI mean 50 | NDBI mean 100 | NDBI mean 500 | NDBI mean 1000 | NDBI mean 2000 | Min temp | Max temp | Mean temp | Daytime mean temp | Temp range | Min humidity | Max humidity | Mean humidity | Daytime mean humidity | Humidity range | Elev meters | Canopy dens | Palms | Mat trees | TS leaflit% | TS soil% | TS palmfr% | TS grass% | TS sand% | TS otherveg% | Aspect deg | Slope deg |
| --- | --- | --- | --- | --- | --- | --- | --- | --- | --- | --- | --- | --- | --- | --- | --- | --- | --- | --- | --- | --- | --- | --- | --- | --- | --- | --- | --- | --- | --- | --- | --- | --- | --- |
| NDVI val |  |  |  |  |  |  |  |  |  |  |  |  |  |  |  |  |  |  |  |  |  |  |  |  |  |  |  |  |  |  |  |  |  |
| NDBI val | -0.67 (<0.0001) |  |  |  |  |  |  |  |  |  |  |  |  |  |  |  |  |  |  |  |  |  |  |  |  |  |  |  |  |  |  |  |  |
| Dist low NDBI | -0.23 (0.0003) | 0.28 (<0.0001) |  |  |  |  |  |  |  |  |  |  |  |  |  |  |  |  |  |  |  |  |  |  |  |  |  |  |  |  |  |  |  |
| Dist med NDBI | 0.55 (<0.0001) | -0.68 (<0.0001) | -0.31 (<0.0001) |  |  |  |  |  |  |  |  |  |  |  |  |  |  |  |  |  |  |  |  |  |  |  |  |  |  |  |  |  |  |
| Dist high NDBI | 0.56 (<0.0001) | -0.59 (<0.0001) | -0.24 (0.0003) | 0.88 (<0.0001) |  |  |  |  |  |  |  |  |  |  |  |  |  |  |  |  |  |  |  |  |  |  |  |  |  |  |  |  |  |
| NDBI max. | -0.56 (<0.0001) | 0.84 (<0.0001) | 0.26 (<0.0001) | -0.84 (<0.0001) | -0.80 (<0.0001) |  |  |  |  |  |  |  |  |  |  |  |  |  |  |  |  |  |  |  |  |  |  |  |  |  |  |  |  |
| NDBI mean 50 | -0.60 (<0.0001) | 0.90 (<0.0001) | 0.28 (<0.0001) | -0.82 (<0.0001) | -0.76 (<0.0001) | 0.96 (<0.0001) |  |  |  |  |  |  |  |  |  |  |  |  |  |  |  |  |  |  |  |  |  |  |  |  |  |  |  |
| NDBI mean 100 | -0.54 (<0.0001) | 0.80 (<0.0001) | 0.25 (<0.0001) | -0.83 (<0.0001) | -0.81 (<0.0001) | 0.95 (<0.0001) | 0.96 (<0.0001) |  |  |  |  |  |  |  |  |  |  |  |  |  |  |  |  |  |  |  |  |  |  |  |  |  |  |
| NDBI mean 500 | -0.53 (<0.0001) | 0.64 (<0.0001) | 0.19 (0.0041) | -0.81 (<0.0001) | -0.84 (<0.0001) | 0.83 (<0.0001) | 0.81 (<0.0001) | 0.87 (<0.0001) |  |  |  |  |  |  |  |  |  |  |  |  |  |  |  |  |  |  |  |  |  |  |  |  |  |
| NDBI mean 1000 | -0.53 (<0.0001) | 0.57 (<0.0001) | 0.18 (0.0045) | -0.85 (<0.0001) | -0.88 (<0.0001) | 0.77 (<0.0001) | 0.74 (<0.0001) | 0.79 (<0.0001) | 0.91 (<0.0001) |  |  |  |  |  |  |  |  |  |  |  |  |  |  |  |  |  |  |  |  |  |  |  |  |
| NDBI mean 2000 | -0.54 (<0.0001) | 0.45 (<0.0001) | 0.13 (0.0477) | -0.77 (<0.0001) | -0.88 (<0.0001) | 0.69 (<0.0001) | 0.66 (<0.0001) | 0.71 (<0.0001) | 0.81 (<0.0001) | 0.90 (<0.0001) |  |  |  |  |  |  |  |  |  |  |  |  |  |  |  |  |  |  |  |  |  |  |  |
| Min temp | -0.03 (0.64) | 0.09 (0.18) | -0.02 (0.74) | -0.10 (0.15) | -0.02 (0.76) | 0.10 (0.15) | 0.10 (0.15) | 0.10 (0.15) | 0.05 (0.43) | 0.05 (0.48) | 0.05 (0.45) |  |  |  |  |  |  |  |  |  |  |  |  |  |  |  |  |  |  |  |  |  |  |
| Max temp | -0.24 (0.0003) | 0.28 (<0.0001) | 0.07 (0.32) | -0.31 (<0.0001) | -0.24 (0.0004) | 0.32 (<0.0001) | 0.33 (<0.0001) | 0.31 (<0.0001) | 0.25 (0.0003) | 0.24 (0.0004) | 0.22 (0.0014) | 0.35 (<0.0001) |  |  |  |  |  |  |  |  |  |  |  |  |  |  |  |  |  |  |  |  |  |
| Mean temp | -0.17 (0.0158) | 0.19 (0.0045) | 0.03 (0.65) | -0.25 (0.0003) | -0.18 (0.0087) | 0.24 (0.0004) | 0.24 (0.0004) | 0.25 (0.0002) | 0.18 (0.0072) | 0.20 (0.0039) | 0.19 (0.0058) | 0.77 (<0.0001) | 0.72 (<0.0001) |  |  |  |  |  |  |  |  |  |  |  |  |  |  |  |  |  |  |  |  |
| Daytime mean temp | -0.19 (0.0046) | 0.20 (0.0032) | 0.02 (0.74) | -0.27 (<0.0001) | -0.20 (0.0030) | 0.26 (0.0001) | 0.26 (0.0001) | 0.26 (0.0001) | 0.20 (0.0028) | 0.22 (0.0014) | 0.20 (0.0039) | 0.61 (<0.0001) | 0.84 (<0.0001) | 0.93 (<0.0001) |  |  |  |  |  |  |  |  |  |  |  |  |  |  |  |  |  |  |  |
| Temp range | -0.24 (0.0005) | 0.25 (0.0003) | 0.09 (0.19) | -0.28 (<0.0001) | -0.24 (0.0004) | 0.29 (<0.0001) | 0.29 (<0.0001) | 0.28 (<0.0001) | 0.22 (0.0010) | 0.22 (0.0014) | 0.20 (0.0034) | -0.02 (0.75) | 0.91 (<0.0001) | 0.46 (<0.0001) | 0.64 (<0.0001) |  |  |  |  |  |  |  |  |  |  |  |  |  |  |  |  |  |  |
| Min humidity | 0.27 (<0.0001) | -0.34 (<0.0001) | -0.03 (0.70) | 0.38 (<0.0001) | 0.34 (<0.0001) | -0.40 (<0.0001) | -0.40 (<0.0001) | -0.40 (<0.0001) | -0.34 (<0.0001) | -0.32 (<0.0001) | -0.29 (<0.0001) | -0.19 (0.0060) | -0.79 (<0.0001) | -0.56 (<0.0001) | -0.70 (<0.0001) | -0.77 (<0.0001) |  |  |  |  |  |  |  |  |  |  |  |  |  |  |  |  |  |
| Max humidity | 0.16 (0.0193) | -0.08 (0.24) | 0.002 (0.97) | 0.09 (0.15) | 0.14 (0.0463) | -0.08 (0.28) | -0.09 (0.19) | -0.10 (0.14) | -0.12 (0.09) | -0.10 (0.13) | -0.15 (0.0318) | -0.20 (0.0035) | -0.24 (0.0004) | -0.32 (<0.0001) | -0.35 (<0.0001) | -0.18 (0.0106) | 0.36 (<0.0001) |  |  |  |  |  |  |  |  |  |  |  |  |  |  |  |  |
| Mean humidity | 0.27 (<0.0001) | -0.27 (<0.0001) | 0.01 (0.86) | 0.35 (<0.0001) | 0.34 (<0.0001) | -0.34 (<0.0001) | -0.33 (<0.0001) | -0.35 (<0.0001) | -0.31 (<0.0001) | -0.32 (<0.0001) | -0.32 (<0.0001) | -0.28 (<0.0001) | -0.67 (<0.0001) | -0.63 (<0.0001) | -0.72 (<0.0001) | -0.61 (<0.0001) | 0.87 (<0.0001) | 0.56 (<0.0001) |  |  |  |  |  |  |  |  |  |  |  |  |  |  |  |
| Daytime mean humidity | 0.27 (<0.0001) | -0.27 (<0.0001) | 0.002 (0.98) | 0.36 (<0.0001) | 0.34 (<0.0001) | -0.34 (<0.0001) | -0.34 (<0.0001) | -0.35 (<0.0001) | -0.31 (<0.0001) | -0.32 (<0.0001) | -0.31 (<0.0001) | -0.27 (<0.0001) | -0.71 (<0.0001) | -0.65 (<0.0001) | -0.76 (<0.0001) | -0.65 (<0.0001) | 0.89 (<0.0001) | 0.50 (<0.0001) | 0.98 (<0.0001) |  |  |  |  |  |  |  |  |  |  |  |  |  |  |
| Humidity range | -0.27 (<0.0001) | 0.34 (<0.0001) | 0.02 (0.72) | -0.38 (<0.0001) | -0.35 (<0.0001) | 0.40 (<0.0001) | 0.40 (<0.0001) | 0.40 (<0.0001) | 0.34 (<0.0001) | 0.32 (<0.00010 | 0.29 (<0.0001) | 0.18 (0.0087) | 0.79 (<0.0001) | 0.56 (<0.0001) | 0.69 (<0.0001) | 0.77 (<0.0001) | -0.99 (<0.0001) | -0.33 (<0.0001) | -0.86 (<0.0001) | -0.88 (<0.0001) |  |  |  |  |  |  |  |  |  |  |  |  |  |
| Elev meters | -0.09 (0.17) | 0.07 (0.27) | 0.04 (0.56) | 0.07 (0.29) | 0.04 (0.53) | -0.03 (0.69) | -0.002 (0.97) | -0.05 (0.46) | -0.09 (0.15) | -0.08 (0.25) | -0.02 (0.74) | 0.005 (0.94) | -0.11 (0.10) | -0.11 (0.10) | -0.12 (0.08) | -0.12 (0.07) | 0.06 (0.39) | -0.09 (0.21) | 0.03 (0.69) | 0.05 (0.16) | -0.06 (0.39) |  |  |  |  |  |  |  |  |  |  |  |  |
| Canopy dens | 0.09 (0.16) | -0.13 (0.05) | -0.17 (0.0083) | 0.16 (0.0143) | 0.13 (0.0447) | -0.13 (0.05) | -0.14 (0.0313) | -0.13 (0.0480) | -0.14 (0.0343) | -0.11 (0.09) | -0.12 (0.06) | -0.003 (0.97) | -0.23 (0.0006) | -0.10 (0.14) | -0.14 (0.0475) | -0.24 (0.0005) | 0.23 (0.0008) | 0.12 (0.08) | 0.17 (0.0123) | 0.18 (0.0092) | -0.22 (0.0011) | -0.04 (0.50) |  |  |  |  |  |  |  |  |  |  |  |
| Palms | 0.34 (<0.0001) | -0.41 (<0.0001) | -0.20 (0.0021) | 0.60 (<0.0001) | 0.58 (<0.0001) | -0.54 (<0.0001) | -0.52 (<0.0001) | -0.54 (<0.0001) | -0.57 (<0.0001) | -0.58 (<0.0001) | -0.54 (<0.0001) | -0.0005 (0.94) | -0.37 (<0.0001) | -0.24 (0.0003) | -0.27 (<0.0001) | -0.36 (<0.0001) | 0.32 (<0.0001) | 0.10 (0.16) | 0.28 (<0.0001) | 0.29 (<0.0001) | -0.32 (<0.0001) | 0.19 (0.0040) | 0.23 (0.0004) |  |  |  |  |  |  |  |  |  |  |
| Mat trees | 0.25 (0.0001) | -0.29 (<0.0001) | -0.19 (0.0028) | 0.46 (<0.0001) | 0.41 (<0.0001) | -0.41 (<0.0001) | -0.38 (<0.0001 | -0.40 (<0.0001) | -0.39 (<0.0001) | -0.42 (<0.0001) | -0.34 (<0.0001) | 0.04 (0.60) | -0.35 (<0.0001) | -0.18 (0.0075) | -0.24 (0.0004) | -0.36 (<0.0001) | 0.33 (<0.0001) | 0.13 (0.07) | 0.27 (<0.0001) | 0.27 (<0.0001) | -0.33 (<0.0001) | 0.15 (0.0247) | 0.18 (0.0055) | 0.46 (<0.0001) |  |  |  |  |  |  |  |  |  |
| TS leaflit% | 0.11 (0.09) | -0.13 (0.0401) | -0.06 (0.36) | 0.09 (0.17) | 0.08 (0.25) | -0.11 (0.10) | -0.14 (0.0358) | -0.15 (0.0239) | -0.16 (0.0175) | -0.11 (0.10) | -0.08 (0.21) | 0.04 (0.59) | 0.01 (0.89) | 0.06 (0.41) | 0.09 (0.19) | 0.01 (0.91) | -0.03 (0.63) | -0.04 (0.53) | -0.7 (0.32) | -0.07 (0.29) | 0.04 (0.56) | -0.06 (0.40) | 0.21 (0.0013) | 0.04 (0.57) | 0.11 (0.09) |  |  |  |  |  |  |  |  |
| TS soil% | 0.09 (0.16) | -0.14 (0.0379) | -0.04 (0.57) | 0.17 (0.0089) | 0.17 (0.0106) | -0.14 (0.0285) | -0.14 (0.0335) | -0.12 (0.07) | -0.07 (0.28) | -0.11 (0.09) | -0.10 (0.14) | -0.11 (0.12) | -0.24 (0.0005) | -0.22 (0.0013) | -0.24 (0.0006) | -0.20 (0.0031) | 0.19 (0.0052) | 0.05 (0.45) | 0.16 (0.0191) | 0.18 (0.0096) | -0.20 (0.0036) | 0.09 (0.18) | -0.05 (0.46) | 0.19 (0.0043) | 0.10 (0.12) | -0.69 (<0.0001) |  |  |  |  |  |  |  |
| TS palmfr% | 0.10 (0.12) | -0.05 (0.47) | -0.03 (0.65) | 0.08 (0.21) | 0.09 (0.16) | -0.02 (0.72) | -0.04 (0.54) | -0.04 (0.59) | -0.06 (0.34) | -0.13 (0.05) | -0.09 (0.17) | -0.08 (0.24) | -0.03 (0.65) | -0.08 (0.21) | -0.07 (0.34) | -0.002 (0.98) | 0.05 (0.52) | 0.08 (0.23) | 0.07 (0.28) | 0.06 (0.42) | -0.04 (0.54) | 0.005 (0.94) | -0.004 (0.95) | 0.13 (0.0395) | -0.06 (0.35) | -0.14 (0.0295) | 0.17 (0.0092) |  |  |  |  |  |  |
| TS grass% | -0.9 (0.18) | 0.15 (0.0201) | 0.06 (0.38) | -0.20 (0.0022) | -0.17 (0.0089) | 0.19 (0.0041) | 0.19 (0.0029) | 0.21 (0.0015) | 0.21 (0.0014) | 0.21 (0.0016) | 0.19 (0.0042) | -0.02 (0.82) | 0.19 (0.0048) | 0.05 (0.44) | 0.07 (0.32) | 0.19 (0.0057) | -0.07 (0.31) | 0.06 (0.36) | 0.03 (0.65) | 0.02 (0.77) | 0.07 (0.34) | -0.05 (0.47) | -0.17 (0.0098) | -0.20 (0.0022) | -0.22 (0.0009) | -0.33 (<0.0001) | -0.7 (0.26) | -0.04 (0.53) |  |  |  |  |  |
| TS sand% | 0.05 (0.40) | -0.07 (0.28) | -0.09 (0.15) | -0.03 (0.64) | -0.01 (0.84) | -0.05 (0.45) | -0.08 (0.20) | -0.06 (0.40) | 0.02 (0.76) | 0.08 (0.23) | 0.07 (0.29) | 0.005 (0.94) | -0.04 (0.52) | -0.02 (0.78) | -0.03 (0.69) | -0.06 (0.35) | -0.004 (0.95) | -0.07 (0.29) | -0.07 (0.29) | -0.05 (0.48) | 0.005 (0.94) | -0.16 (0.0176) | -0.10 (0.15) | 0.002 (0.98) | -0.11 (0.09) | -0.17 (0.0114) | 0.13 (0.0481) | -0.02 (0.77) | -0.4 (0.58) |  |  |  |  |
| TS otherveg% | -0.25 (<0.0001) | 0.31 (<0.0001) | 0.15 (0.0191) | -0.31 (<0.0001) | -0.30 (<0.0001) | 0.29 (<0.0001) | 0.32 (<0.00010 | 0.32 (<0.0001) | 0.25 (0.0001) | 0.24 (0.0002) | 0.21 (0.0010) | 0.23 (0.0009) | 0.22 (0.0010) | 0.26 (<0.0001) | 0.19 (0.0052) | 0.15 (0.0323) | -0.19 (0.0060) | -0.45 (0.52) | -0.16 (0.0188) | -0.17 (0.0132) | 0.19 (0.0064) | 0.04 (0.56) | -0.16 (0.0146) | -0.21 (0.0012) | -0.08 (0.21) | -0.32 (<0.0001) | -0.25 (<0.0001) | -0.08 (0.20) | 0.09 (0.18) | -0.08 (0.25) |  |  |  |
| Aspect deg | 0.04 (0.52) | 0.13 (0.0391) | 0.006 (0.93) | 0.02 (0.72) | 0.05 (0.46) | 0.10 (0.14) | 0.12 (0.06) | 0.11 (0.09) | 0.02 (0.71) | -0.01 (0.87) | -0.03 (0.70) | -0.06 (0.41) | 0.01 (0.84) | -0.01 (0.87) | 0.002 (0.97) | 0.01 (0.79) | 0.03 (0.66) | -0.05 (0.46) | 0.03 (0.64) | 0.04 (0.55) | -0.03 (0.66) | -0.3 (0.66) | 0.08 (0.24) | -0.02 (0.71) | 0.05 (0.48) | 0.05 (0.44) | -0.05 (0.49) | -0.05 (0.41) | -0.05 (0.49) | -0.10 (0.15) | 0.01 (0.85) |  |  |
| Slope deg | 0.10 (0.85) | -0.11 (0.09) | -0.05 (0.46) | 0.21 (0.0014) | 0.14 (0.0373) | -0.17 (0.0085) | -0.16 (0.0152) | -0.16 (0.0168) | -0.11 (0.11) | -0.13 (0.0494) | -0.05 (0.43) | 0.04 (0.54) | -0.002 (0.98) | 0.01 (0.86) | 0.004 (0.96) | -0.04 (0.60) | -0.02 (0.82) | 0.004 (0.95) | -0.01 (0.88) | -0.03 (0.71) | 0.02 (0.83) | -0.002 (0.98) | 0.04 (0.52) | 0.19 (0.0035) | 0.12 (0.08) | -0.15 (0.0196) | 0.19 (0.0042) | 0.04 (0.52) | -0.4 (0.55) | 0.16 (0.0152) | -0.03 (0.65) | 0.02 (0.77) |  |

**NDVI val** = NDVI value of sampled site; **NDBI val** = NDBI value of sampled site; **Dist low NDBI** = Distance (meters) to the nearest low NDBI pixel; **Dist med NDBI** = Distance (meters) to the nearest medium NDBI pixel; **Dist high NDBI** = Distance (meters) to the nearest high NDBI pixel; **NDBI max.** = Maximum NDBI in 3 x 3 pixel neighborhood coinciding with the point; **NDBI mean 50** = Mean NDBI value within a 50 m buffer zone surrounding the point; **NDBI mean 100** = Mean NDBI value within a 100 m buffer zone surrounding the point; **NDBI mean 500** = Mean NDBI value within a 500 m buffer zone surrounding the point; **NDBI mean 1000** = Mean NDBI value within a 1000 m buffer zone surrounding the point; **NDBI mean 2000** = Mean NDBI value within a 2000 m buffer zone surrounding the point; **Min temp** = Minimum temp (°C) recorded during the sampling period; **Max temp** = Maximum temp (°C) recorded during the sampling period; **Mean temp** = Mean temp (°C) recorded during the sampling period; **Daytime mean temp** = Mean temp (°C) recorded during daytime hours (06:00 - 18:00); **Temp range** = Difference between minimum and maximum temp (°C) recorded during the sampling period; **Min humidity** = Minimum relative humidity (%) recorded during the sampling period; **Max humidity** = Maximum relative humidity (%) recorded during the sampling period; **Mean humidity** = Mean relative humidity (%) recorded during the sampling period; **Daytime mean humidity** = Mean relative humidity (%) recorded during daytime hours (06:00 - 18:00); **Humidity range** = Difference between minimum and maximum relative humidity (%) recorded during the sampling period; **Elev meters** = Elevation in meters; **Canopy dens** = Canopy cover (%) estimated using densiometer; **Palms** = Number of palms within 5m radius of trap; **Mat trees** = Number of mature trees within 5m radius of trap; **TS leaflit%** = Trap substrate: % leaf litter directly beneath trap; **TS soil%** = Trap substrate: % soil directly beneath trap; **TS palmfr%** = Trap substrate: % palm frond directly beneath trap; **TS grass%** = Trap substrate: % grass directly beneath trap; **TS sand%** = Trap substrate: % sand directly beneath trap; **TS otherveg%** = Trap substrate: % other vegetation directly beneath trap; **Aspect deg** = Aspect at collection site (degrees); **Slope deg** = Slope at collection site (degrees).
