# Supplementary material for: Where boundaries become bridges: Mosquito community composition, key vectors, and environmental associations at forest edges in the central Brazilian Amazon
Source: PLoS Negl Trop Dis. 2023 Apr 26;17(4):e0011296. doi: 10.1371/journal.pntd.0011296 (PMC10166490; doi:10.1371/journal.pntd.0011296)
Supplement: S4 Table — (DOCX) [file pntd.0011296.s005.docx]

| **Distance** | **Elevation** (m) | **NDVI value** | **Average ground cover value*** | **Number of palms** | **Number of mature trees** | **Maximum temperature (°C)** |
| --- | --- | --- | --- | --- | --- | --- |
| **0 m** | 76.81 (1.87) | 0.73 (0.015) | 2.03 (0.06) | 2.25 (0.28) | 0.95 (0.10) | 30.42 (0.40) |
| **500 m** | 91.60 (3.40) | 0.86 (0.002) | 1.81 (0.08) | 6.62 (0.61) | 2.29 (0.25) | 27.94 (0.23) |
| **1000 m** | 82.03 (3.01) | 0.86 (0.002) | 1.59 (0.10) | 6.42 (0.52) | 2.58 (0.27) | 28.00 (0.28) |
| **2000 m** | 80.29 (2.59) | 0.87 (0.002) | 1.49 (0.08) | 8.55 (0.80) | 2.71 (0.28) | 28.00 (0.26) |

**S4 Table.** Mean values (± 1 standard error) at each distance for the six specified environmental variables included in analyses of the occurrence of key mosquito taxa.

*****Percent ground vegetation cover expressed as a value.
