# Supplementary material for: Where boundaries become bridges: Mosquito community composition, key vectors, and environmental associations at forest edges in the central Brazilian Amazon
Source: PLoS Negl Trop Dis. 2023 Apr 26;17(4):e0011296. doi: 10.1371/journal.pntd.0011296 (PMC10166490; doi:10.1371/journal.pntd.0011296)
Supplement: S5 Table — (DOCX) [file pntd.0011296.s006.docx]

**S5 Table.** Mean distance in meters ± 1 standard error (S.E.) of positive sites to low, medium, and high NDBI pixels for key taxa.

| **Species** | **Number of edge sites in which taxa were detected** | **Mean distance (m) to low NDBI (± 1 S.E.)** | **Mean distance (m) to medium NDBI (± 1 S.E.)** | **Mean distance (m) to high NDBI (± 1 S.E.)** |
| --- | --- | --- | --- | --- |
| *Aedes aegypti* | 3 | 15.19 (4.89) | 20.01 (2.93) | 48.24 (3.80) |
| *Aedes albopictus* | 65 | 14.56 (1.04) | 36.46 (3.94) | 153.74 (23.88) |
| *Sabethes* spp.* | 22 | 15.04 (2.15) | 74.17 (22.44) | 339.87 (68.70) |
| *Haemagogus* spp.* | 6 | 11.56 (2.44) | 58.29 (18.75) | 185.17 (66.4) |
| *Psorophora* spp.* | 50 | 15.26 (1.33) | 54.78 (10.02) | 218.03 (34.11) |

*Grouped at genus-level.
